# Profile of Bioactive Compounds, Aromas, and Cup Quality of Excelsa Coffee (*Coffea liberica* var. *dewevrei*) Prepared from Diverse Postharvest Processes

**DOI:** 10.1155/2022/2365603

**Published:** 2022-08-18

**Authors:** Dian Herawati, Michael Oscarius Loisanjaya, Radwa Husni Kamal, Dede Robiatul Adawiyah, Nuri Andarwulan

**Affiliations:** ^1^Department of Food Science and Technology, Faculty of Agricultural Engineering and Technology, IPB University, Bogor, Indonesia; ^2^South-East Asia Food and Agricultural Science and Technology Center, IPB University, Bogor, Indonesia

## Abstract

The study is aimed at evaluating bioactive compounds, volatile compounds, and cup quality of Excelsa coffee (*Coffea liberica* var. *dewevrei*) from different postharvest processing procedures, i.e., natural, honey, semiwashed, and wine. The green beans from each procedure were roasted at light to medium levels. Sample analysis was performed using HPLC and GC-MS instruments for bioactive compounds and volatile compounds, respectively, followed by a cupping test. As the results, postharvest processing significantly altered content of bioactive compounds (caffeoylquinic acids (CQAs) and alkaloids) in Excelsa green beans; the lowest quantity of CQAs and alkaloids was found in wine and semiwashed green beans, respectively. Significant degradation of 5-CQA and its transformation to 3-CQA and 4-CQA occurred in all light-medium roasting levels. In general, alkaloids were stable during roasting, and only trigonelline was slightly declined. Roasting process also generated 17 potent volatile compounds in Excelsa beans including 2-ethylpyrazine, 2,3-dimethylpyrazine, 2,5-dimethylpyrazine, 2,5-dimethyl-3-ethylpyrazine, guaiacol, 4-vinyl-guaiacol, and isovaleric acid and its esters. Furthermore, cupping test revealed that Excelsa coffee samples obtained from different postharvest processing were categorized as very good coffee (cupping score > 80). This finding may encourage the commercialization of all observed Excelsa coffee beans.

## 1. Introduction

For centuries, coffee has been a popular beverage due to its unique flavor and functional properties as the bean contains various chemical compounds. Superior coffee quality is often described with a pleasant sensation and a balanced combination of flavor with body and aroma as well as in the absence of faults. Zapata et al. [1] revealed that chemical compounds of coffee, such as 2-methylbutanal, 2-methyl-2-butenal, 2-furancarboxaldehyde, furfural, and methyl phenylacetate, accounted for the highest sensory activities in roasted coffee, suggesting that these chemicals are crucial for coffee flavor formation. In addition, the chemical compounds in coffee bean are reported to exert beneficial effects on human health. Some significant compounds of coffee included chlorogenic acids (caffeoylquinic acids, di-caffeoylquinic acids, and feruloyl-quinic acids), caffeine, cafestol, and kahweol [2].

Liberica coffee (*Coffea liberica*) is one of identified coffee species, despite less popular in market compared to *Coffea arabica* L. (Arabica coffee) and *Coffea canephora* P. (Robusta coffee) [3]. Liberica exists in two varieties, i.e., *Coffea liberica* var. *liberica* and *Coffea liberica* var. *dewevrei*; the latter is commercially known as Excelsa coffee. In many cases, Excelsa coffee is perceived by coffee lovers as unique flavor-rich beverage.

Relation between food chemistry and flavor profile of coffee is complex. The complexity relates to numerous factors affecting flavor and bioactive components of coffee bean, such as postharvest processing, roasting degree, brewing method, and geographical condition of coffee plantation [4, 5]. These factors are responsible for generation of precursor compound present in coffee beans, which in turn differentiates sensory properties. Postharvest processing of coffee cherries into green bean can be conducted by numerous methods, namely, semiwash, full-wash, honey, wine, and natural [6]. The difference occurs in the washing step, drying time, and presence of fermentation. As reported by Abubakar et al. [7], the methods resulted in different sensory characteristics, especially fragrance remarks of coffee brew. This finding exhibits that the bean receives different chemical changes between procedures.

Furthermore, roasting process prompts production of volatile compounds at more intensive degree, which links to a formation of unique taste on coffee brew. In this stage, Maillard reaction occurred as induced by heat exposure, producing melanoidin and heterocyclic components. Intriguingly, current studies revealed that both components contributed to formation of coffee flavor and antioxidant activity [8, 9].

Excelsa coffee belongs to a liberica species, mostly grown in Southeast Asia and merely accounts for 2% of world coffee consumption. Despite many studies discussing chemical composition and sensory evaluation of Excelsa coffee, they are unable to cover substantial insight on how postharvest processes affect chemical profile. Therefore, extensive investigation of the Excelsa coffee is needed to explore the effect of various postharvest processing methods on the chemical composition (volatile and bioactive compounds), as well as sensory quality (cupping score). To fill the gap, this study is aimed at characterizing volatile and bioactive components and sensory quality of Excelsa coffee from different processing conditions. The attributes observed in the high sensory quality of Excelsa coffee are expected to be used as key indicators to select coffee processing for commercial purposes.

## 2. Materials and Methods

### 2.1. Chemicals

Chemicals such as HPLC grade water, HPLC grade methanol, formic acid, CQAs standard (3-CQA, 4-CQA, and 5-CQA), and alkaloids standard (trigonelline, theobromine, and caffeine) were purchased from Merck (Darmstadt, Germany). Other chemicals were analytical grade purchased from selected suppliers.

### 2.2. Sample Preparation

**Figure 1:**
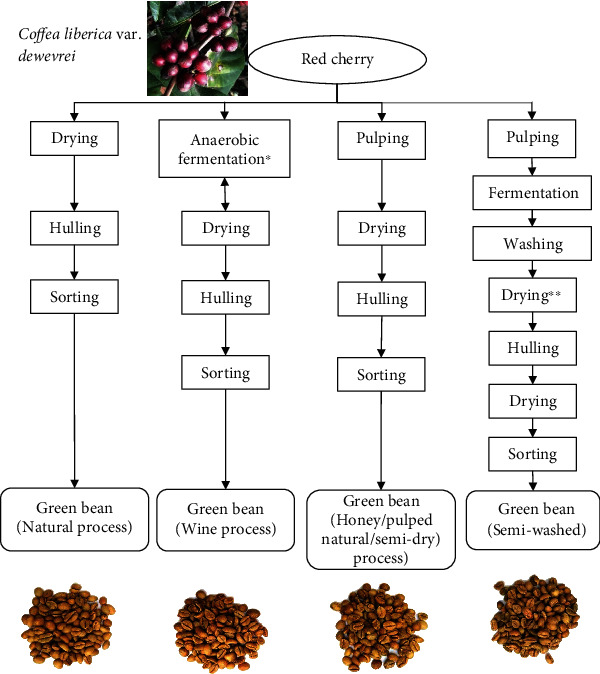
Main steps for different postharvest processing of Excelsa coffee (Coffea liberica var. dewevrei). ^∗^The anaerobic condition is intercepted for the drying process repeatedly until the farmers get the desired moisture content. ^∗∗^Initial drying reaches moisture content approximately 40%.

Excelsa coffee beans were collected from Wonosalam in Province of East Java. They were treated differently by local farmers as follows: natural, wine, honey, and semiwashed, as presented in Figure 1. Natural processed beans (3 samples) were supplied from different farmers (farmers 1, 2, and 3), while wine, honey, and semiwashed beans were collected from farmer 1.

Excelsa green beans (30 g) were crushed using a household mill, after treated with liquid nitrogen. The grinding process was conducted after 5 minutes elapsed and done for 1 minute to obtain a fine green bean powder.

Meanwhile, to obtain roasted coffee beans, a roaster machine (Garuda Roaster, Indonesia) with a maximum capacity of 1 kilogram was used. The initial temperature was set at 200°C, while the final temperature was 220°C. Roasting process was performed for 12 minutes, classified as light to a medium roasting degree. The lightness value (*L*∗) of roasted beans was 37-38, measured using chromameter (CR-400, Konica Minolta, Osaka, Japan). The roasted coffee powder was obtained by grinding the beans using a coffee grinder Latina 600 N (Yang Chia Machine Works, Taiwan) with the highest grinding level, yielding fine coffee powder. The coffee powder was packed in aluminum foil packaging and stored in the freezer for further analysis.

### 2.3. Bioactive Compound Analysis

Green and roasted coffee powder was extracted by using percolation [10]. The coffee extract (20 *μ*L) was injected into HPLC equipped with UV-Vis detector (LC-20AD system; SHIMADZU Corp., Kyoto, Japan). For CQA content analysis, a method developed by Herawati et al. [11] was used without any modification. For calibration and linearity, standard curves of CQA compound were obtained from 6 points of 3-CQA, 4-CQA, and 5-CQA compounds, with concentration ranging from 16 to 500 mg/L (triplicate; LoD of 3-CQA = 13.27 mg/L with *R*^2^ = 0.997, LoD of 4-CQA = 15.37 mg/L with *R*^2^ = 0.996, and LoD of 5-CQA = 17.16 mg/L with *R*^2^ = 0.996).

Quantification of alkaloid followed the procedure of Caprioli et al. [12] with minor modification. Elution of alkaloids in Excelsa coffee brew was performed in Zorbax C18 column (id. 4.6 × 150 mm, 5 *μ*m). The eluent was methanol LC grade (A) and formic acid 0.3% (B), with a flow rate of 0.4 mL/min. The gradient was set as follows: 25% A (0 min), 60% A (0-10 min), 60% A (10-15 min), 25% A (15-20 min), and 25% A (20-25 min), with detection wavelength at 265 nm. For alkaloid calibration, a 6-point standard curve of mixed trigonelline, theobromine, and caffeine was used, with concentration ranging from 6 to 200 mg/L (triplicate; LoD of trigonelline = 1.08 mg/L with *R*^2^ = 0.999, LoD of theobromine = 2.40 mg/L with *R*^2^ = 0.999, and LoD of caffeine = 0.93 mg/L with *R*^2^ = 0.999).

### 2.4. Volatile Compound Analysis

Green and roasted coffee beans were extracted using SPME (Solid Phase Micro Extraction) method. The coffee powder (5 g for roasted samples) and (7 g for green bean samples) was added with internal standard (3-heptanone, 5 *μ*L for roasted coffee powder, and 3 *μ*L for green coffee powder) with 0.01% concentration in HPLC grade methanol. The mixture was transferred into a 22 mL vial SPME for extraction performed for 30 minutes at 50°C with SPME fiber DVB/CAR/PDMS 2 cm [13, 14]. Subsequently, the mixture was injected automatically into GC-MS instrument (GC Agilent 7890A, California, United States). The elution was performed in a DB-Wax column (30 m × 250 *μ*m × 0.25 *μ*m) equipped with MS Agilent 595975C (triple-axis detector). The carrier gas (helium) was run at a flow rate of 1 mL/minute and injection temperature of 250°C. The oven setting was set as follows: 60°C for 0 min, 4°C/min to 120°C for 0 min, and then 6°C/min to 240°C for 10 min.

Profile of volatile compounds was identified in National Institute of Standards and Technology (NIST) 14 library software, by comparing mass spectra of compounds in the samples with mass spectra library of the NIST 14 collection. Moreover, linear retention index (LRI) of each chemical compound was determined according to retention time of a standard series of alkanes.

Furthermore, to confirm LRI value, data from this experiment were compared with the LRI value of the same compound using the same method and similar column in published references. Relative concentration of volatile compounds was also calculated by comparing peak area of the specific compound in the sample with the peak area of the 3-heptanone internal standard (0.01% *w*/*v*) added to the sample before the extraction process.

### 2.5. Sensory Evaluation

Ethical clearance for sensory evaluation was approved by the research ethics commission number 297/IT3.KEPMSM-IPB/SK/2020. The sensory evaluation was carried out via cupping tests by three trained coffee expert/Q-graders [6, 15, 16]. The procedure conformed to Specialty Coffee Association of America (SCAA) protocol [17].

### 2.6. Statistical Analysis

Data were tested statistically using ANOVA in the SPSS 22 software (IBM SPSS Statistics 22, USA). Significant difference between means was compared using Duncan test at *P* < 0.05. Moreover, discrimination among green bean samples also was analyzed using principal component analysis (PCA) in the XLSTAT software version 2021.1 (Addinsoft, Paris, France).

## 3. Results and Discussion

### 3.1. Bioactive Profile of Excelsa Coffee Brew

**Figure 2:**
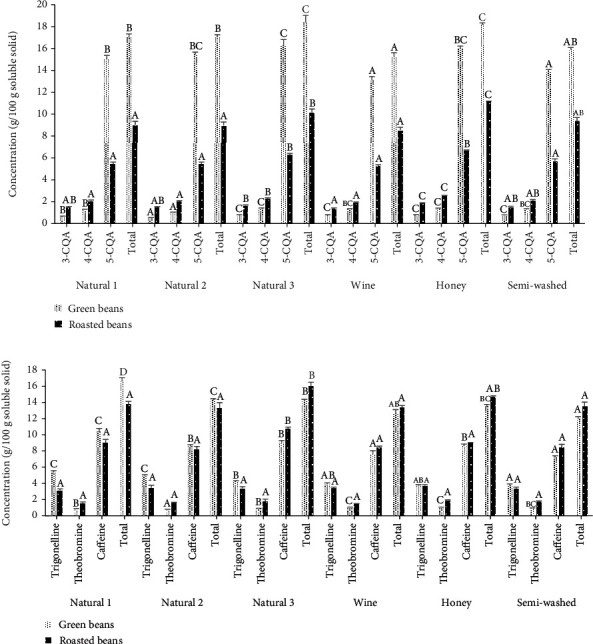
Caffeoylquinic acid (a) and alkaloid (b) composition of green and roasted bean extract of Excelsa coffee obtained from different processes. The value displayed in the figure is the average and standard error of three replications. ANOVA test on green and roasted coffee beans was carried out separately. Different superscripts indicate differences among samples (*P* < 0.05).

CQAs (especially 3-CQA, 4-CQA, and 5-CQA) and alkaloids (caffeine, trigonelline, and theobromine) are key bioactive compounds in coffee. In this work, we reported their concentration present in Excelsa coffee brew processed using different postharvest processes (Figure 2).

The results showed significant effects of postharvest treatments on the concentration of CQAs and alkaloids of Excelsa green beans (*P* < 0.05). In general, the highest concentration of CQAs was found in honey green bean sample, while the lowest was found in wine bean sample. Honey process, also known as pulped natural or semidry process, involved cherry pulping, while its bean mucilage was purposely retained at diverse degree. The pulped cherry was sun-dried directly. Duarte et al. [18] found that honey process in Arabica coffee significantly resulted in a higher amount of CQAs (3-CQA, 4-CQA, and 5-CQA) compared to dry (natural) process. The higher retention of CQAs in honey-processed beans was caused by a shorter drying time. Pulp removal in honey process enables to suppress drying time, as reported by Poltronieri and Rossi [19], lowering chemical degradation. In addition, honey process was performed without washing stage; therefore, it prevented CQA leaching from the bean. Meanwhile, in semiwash process, pulping was followed by fermentation and washing, which increased the release of CQAs.

Regarding the comparison of dry and wine process, we reported an unexpected result. Since wine process constitutes a modification of the dry (natural) process, content of CQAs in wine process should be similar to that in dry process. Intriguingly, a lower concentration of 5-CQA was observed in wine sample. The dissimilarity may be caused by the longer drying time in wine process, which prompts a more intensive degradation of 5-CQA.

In terms of alkaloids, the highest caffeine content of green coffee brew was found in natural and honey samples. Meanwhile, honey and wine samples were found to have the highest theobromine content. Caffeine content in wine samples was significantly lower than that in natural samples. The results seem to confirm that the more intensive sun-drying process leads to a higher degradation of caffeine in green coffee beans. On the other hand, caffeine content in honey sample was significantly higher than that in semiwashed sample. This result is concordant with a previous research reporting that the honey-processed coffee bean contains a significantly higher quantity of caffeine compared to the wet-processed coffee bean [20]. Another research also supports our present study, confirming that wet treatment results in a lower content of caffeine and trigonelline in comparison with dry treatment [21].

**Figure 3:**
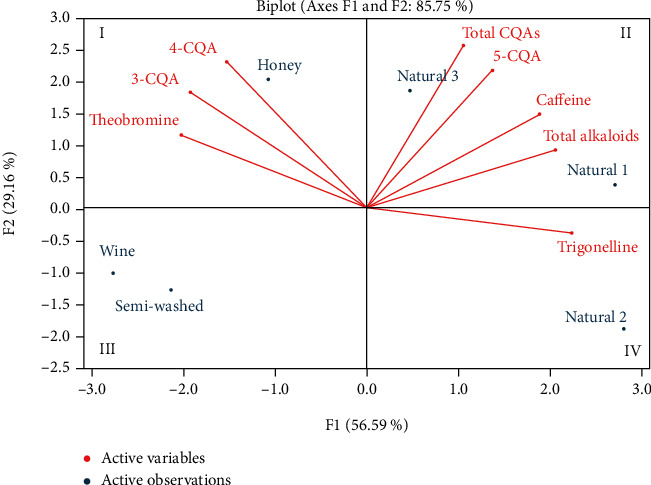
Biplot of caffeoylquinic acid (CQA) and alkaloid composition of green bean extract of Excelsa coffee obtained from different processes. Natural 1, 2, and 3 samples were from different farmers. Natural 1, wine, honey, and semiwashed samples were from same farmer. The value displayed in the figure is the average of three replications.

In order to emphasize the similarity and dissimilarity among the green bean samples, PCA analysis result is presented in Figure 3. Green bean samples were separated in four quadrants. Wine and semiwashed samples were located in the quadrant III associated with low CQAs and alkaloids, whereas high 3-CQA, 4-CQA, and theobromine were found in honey sample, sitting in the quadrant I. Regarding to natural samples, there was variation among samples. In general, natural sample position for CQA and alkaloid concentration was in the second order after honey sample.

Roasting process markedly altered content of CQAs. After roasting, 5-CQA was significantly lower; in contrast, quantity of 3-CQA and 4-CQA doubled following the roasting (Figure 2). This consistently occurs in all coffee samples. Moon et al. [22] reported that light roasting of Ethiopian, Nicaraguan, and Sumatran coffee samples induced increment of the 4-CQA concentration. It is noteworthy that CQAs can turn into CQA lactones during the roasting stage. However, in some cases, isomerization of CQA may occur before the lactone formation, increasing the content of 3-CQA and 4-CQA [23, 24].

Meanwhile, noticeable degradation of trigonelline was found in our experiment. Trigonelline degradation in natural green bean samples was faster than in the other postharvest groups. It is because natural green beans contained higher trigonelline concentration which was available for degradation reaction. After trigonelline reached certain concentration, the degradation rate may slow down, and at the end, all roasted samples contained similar amount of trigonelline. However, further study is needed to investigate the mechanism of trigonelline degradation during roasting. Previous studies also reported similar results which underline degradation of trigonelline during the roasting process; in this regard, a decrease in trigonelline was followed by increase in nicotinic acid as one of its derivatives [25, 26].

Unlike trigonelline, caffeine experienced a different fate, in which it showed no significant degradation during roasting. The stability of caffeine during roasting was also observed by previous studies [10, 27]. Theobromine in Excelsa coffee was observed to increase approximately 1.8 times higher after the roasting process. Conversely, Jeszka-Skowron et al. [25] reported a decrease of theobromine after the roasting process.

### 3.2. Volatile Compound Profile of Excelsa Coffee Powder

Table 1 and Table 1S (supplementary file) present composition of volatile compound successfully identified using GC-MS. A total of 59 volatile compounds from 12 different classes were detected in Excelsa green coffee beans, while roasted coffee beans showed 85 volatile compounds from 18 chemical classes.

Regarding the chemical classes, volatile compounds in Excelsa roasted beans were more miscellaneous than those in green beans. It is believed that roasting stage facilitates numerous reactions that produce new compounds. Pyrimidine derivatives, benzoxazine, pyrroles, thiophene, pyrone, and quinolone were chemical classes only detected in roasted beans, but they were absent in green beans (Table 1).

Meanwhile, carboxylic acid, ester, aldehyde, and alkane classes were found to dominate among all classes present in Excelsa green beans. These results are in agreement with previous research reporting the abundance of these chemicals in green coffee beans, i.e., alcohol, esters, hydrocarbons, and aldehydes [28]. Additionally, Tsegay et al. [9] reported presence of chemical classes, i.e., furans, pyrazines, and pyridines, in green and roasted coffee. Our present work confirmed the finding, since we also observed these compounds (i.e., pyridine, furan, ketone, pyrazine, and pyrazole) in the green beans.

The results demonstrated that volatile composition of Excelsa green coffee obtained from different postharvest processing was similar, except for wine sample. Joët et al. [29] and Knopp et al. [30] confirmed that metabolic reactions during the drying process could lead to interconversion of low molecular weight sugar and hydrolysis of proteins. In this regard, the investigation pertaining to the effects of drying on the chemical compounds of green coffee beans is limited to exploration of major constituents such as proteins and carbohydrates. However, specific studies discovering volatile compound changes due to the drying process are still rather scarce.

This study found that green beans obtained from wine process contained more carboxylic acid and ester compared to the other samples, because prolonged anaerobic fermentation and drying may facilitate the production of those compounds. Noticeable insights were observed in the roasted wine coffee samples, since they contained less volatile compounds than the other samples. The reactions during wine process markedly reduced coffee aroma precursors as indicated in Figure 2. The major chlorogenic acid (5-CQA) content in wine process was the lowest; on the other hand, it is considered as one of chemical drivers of aroma properties induced by roasting process [13, 31].

Volatile compounds detected in Excelsa roasted beans were also detected in Arabica and Robusta coffee beans [13, 32]. Some of these compounds provide essential contribution to Excelsa roasted bean aroma. This study proposed impact compounds of Excelsa roasted beans, referring to the impact compounds detected in Arabica/Robusta coffee from previous studies. In addition, the compounds with high intensity were also considered as proposed impact compounds. As presented in Table 2, there are 17 proposed impact compounds present in Excelsa coffee. Among them, six compounds (2-ethylpyrazine, 2,3-dimethylpyrazine, 2,5-dimethylpyrazine, 2,5-dimethyl-3-ethylpyrazine, 2-methoxyphenol (guaiacol), and 4-vinyl-2-methoxyphenol (4-vinyl-guaiacol)) are impact compounds of Arabica coffee [32]. The concentration of 4-vinyl-guaiacol and some pyrazines in Robusta are higher than those in Arabica, while 2,3-pentanedione is higher in Arabica [13]. Isovaleric acid and ethyl isovalerate were also contributor to Excelsa aroma compounds. Isovaleric acid gives rancid and cheese aroma, while ethyl isovalerate contributes to fruity aroma [13, 33]. Isovaleric acid and its ester are important volatile compounds in jackfruit [34]. They may contribute to jackfruit aroma, which are commonly detected in Excelsa coffee. In this case, it can be explained the reason why Excelsa coffee aroma is more similar with typical fruity aroma of Arabica rather than Robusta coffee as observed by coffee cupper.

### 3.3. Sensory Properties of Excelsa Coffee

Table 3 presents the cupping score of Excelsa coffee prepared from different postharvest processing. Cupping score test was conducted using a similar method used for Arabica coffee [17], considering that Excelsa coffee samples were similar to Arabica coffee based on the preliminary assessment by coffee cupper as mentioned above.

As the results, all coffee samples are categorized as very good coffee (specialty coffee for Arabica beans) because their score is above 80 [17, 35]. Even though the statistical analysis enables to differentiate the samples, the total score of all samples are quite similar in the range of 82.33–83.83. The significant difference of the scores was found in five attributes: aroma, aftertaste, acidity, body, balance, and overall. Among samples tested, the cupping score for natural beans from farmer 2 was slightly higher in aroma, acidity, balance, and overall attributes. According to observed bioactive and volatile compounds, this sample had a moderate concentration of all compound groups. The balance composition of this sample may be responsible for the high scores of the attributes mentioned above.

The score given by the coffee cupper in the cupping score test is not the absolute rating intensity, but it is affected by subjective appraisal of the individual attribute [36]. However, it is an internationally recognized sensory test to grade the coffee beans for commercial use. This cupping score is still useful to describe general cupping quality of Excelsa coffee position in coffee trading. Further scientific sensory analysis such as Quantitative Descriptive Analysis by trained panelists is needed to evaluate absolute rating intensity of the essential attributes in order to gain the sensory profile of Excelsa coffee.

## 4. Conclusion

CQAs and alkaloids can be a fingerprint of green beans obtained from different postharvest processing. The order of CQA concentration in the sample is honey > natural > semiwashed > wine green beans. Meanwhile, the lowest concentration of alkaloids is found in semiwashed green bean. Excelsa coffee roasting in the light to medium degree facilitates transformation of 5-CQA into 3-CQA and 4-CQA. There are 17 compounds proposed as Excelsa roasted coffee potent aromas including 2-ethylpyrazine, 2,3-dimethylpyrazine, 2,5-dimethylpyrazine, 2,5-dimethyl-3-ethylpyrazine, guaiacol, 4-vinyl-guaiacol, and isovaleric acid and its esters. According to cupping test, all observed Excelsa coffee samples are very good coffee (cupping score > 80). Further scientific sensory analysis is envisaged to understand the sensory profile of Excelsa coffee.

## Figures and Tables

**Table 1:** Volatile compound classes of green and roasted Excelsa coffee powder from different plantation condition and different postharvest processing.

Classes	^∗^Concentration in green beans (*μ*g/kg beans)						∗Concentration in roasted beans (*μ*g/kg beans)					
	Natural 1	Natural 2	Natural 3	Wine	Honey	Semiwashed	Natural 1	Natural 2	Natural 3	Wine	Honey	Semiwashed
Aldehyde	113.2	101.2	102.4	146.4	151.4	115.1	1431.7	1553.9	1304.3	803.7	1782.2	1808.7
Alcohol	47.6	71.5	65.7	75.5	70.5	45.0	2123.4	1792.6	1883.9	890.7	2215.4	2334.3
Carboxylic acid	184.2	194.2	151.1	272.8	271.1	175.4	6587.7	6971.1	6978.2	3951.4	10241.1	7912.7
Ester	128.8	170.3	138.4	262.0	165.3	140.1	249.8	256.3	250.8	171.3	318.2	291.5
Ketone	41.43	44.37	45.95	58.11	48.40	30.71	1495.6	1625.4	1236.3	999.3	1967.5	2187.6
Alkane	73.6	89.7	109.1	127.4	95.6	72.2	119.2	134.8	138.9	52.3	134.5	133.4
Pyridine	7.85	10.77	7.15	8.67	9.99	7.24	2501.2	2872.5	2595.9	1713.0	3025.1	2902.7
Pyrimidine derivative	0.00	0.00	0.00	0.00	0.00	0.00	386.3	336.6	318.1	146.6	454.3	563.4
Furan	3.3	4.3	4.8	5.3	6.3	3.7	3284.7	3586.3	2446.5	2635.3	5335.2	536.0
Pyrazine	18.0	39.6	26.5	33.2	33.2	18.7	5033.9	4684.9	4682.2	2352.4	6192.4	5315.1
Pyrazole	1.0	1.2	1.2	1.0	1.9	1.1	167.8	220.4	116.9	131.1	164.1	278.4
Benzoxazine	0.0	0.0	0.0	0.0	0.0	0.0	54.5	67.3	81.6	32.0	120.8	109.9
Hydrazine	0.0	0.0	0.8	0.0	0.0	0.0	20.4	21.6	23.2	0,00	112.2	21.2
Pyrrole	0.0	0.0	0.0	0.0	0.0	0.0	454.0	419.0	339.1	257.6	588.2	640.9
Thiophene	0.0	0.0	0.0	0.0	0.0	0.0	37.6	51.5	49.0	25.7	50.7	68.7
Pyrone	0.0	0.0	0.0	0.0	0.0	0.0	203.3	242.4	246.1	138.2	268.4	327.3
Quinolone	0.0	0.0	0.0	0.0	0.0	0.0	64.8	86.5	85.2	42.5	73.6	108.0
Benzene	29.1	37.8	25.9	55.2	37.7	10.9	1076.9	1199.9	1060.7	652.3	1311.7	1149.3

LRI: linear retention index; nd: not detected. Natural 1, 2, and 3 samples were from different farmers. Natural 1, wine, honey, and semiwashed samples were from same farmer. ^∗^The relative concentration of volatile compounds was calculated by comparing the peak area of the specific compound in the sample with the peak area of internal standard (3-heptanone). The values displayed in the table are average of two replications.

**Table 2:** Identified potent aromas in roasted Excelsa coffee powder obtained from different postharvest processing.

Compound in roasted beans	Sensory descriptors
Acetic acid	Pungent, vinegar^1^
Hydroxy-acetic acid	—
Isovaleric acid	Rancid, cheesy^2^
Ethyl isovalerate	Fruity^3^
Methylpyrazine	Nutty^1^
2,5-Dimethylpyrazine	Hazelnut/roasted^4^
2-Ethylpyrazine	Peanuts/roasted^4^
2,3-Dimethylpyrazine	Hazelnut/roasted^4^
2,5-Dimethyl-3-ethylpyrazine	Earthy roasted^1,4^
2,6-Dimethylpyrazine	Chocolate, cocoa, roasted nuts, fried^1^
2-Methoxyphenol (guaiacol)	Phenolic, burnt, smoky^1^
4-Vinyl-2-methoxyphenol (4-vinyl-guaiacol)	Phenolic, clove^4^
Pyridine	Sour, putrid, fishy, amine, bitter, roasted^1^
Dihydro-2-methyl-3-furanone	—
2,3-Pentanedione	Buttery, oily, caramel-like^1^
Furfural	Sweet, woody, almond^1^
5-Methylfurfural	Spice, caramel, maple^1^

^1^Caporaso et al. [13]; ^2^Seninde and Chambers [26]; ^3^Gonzalez-Rios et al. [33]; ^4^Toci et al. [32].

**Table 3:** Cupping score of Excelsa coffee brew obtained from different postharvest processing.

Roasted bean samples	Fragrance/aroma	Flavor	Aftertaste	Acidity	Body	Uniformity	Balance	Clean cup	Sweetness	Overall	Total cupping score
Natural 1	7.58 ± 0.14^a^	7.67 ± 0.14^a^	7.58 ± 0.14^b^	7.58 ± 0.14^ab^	7.67 ± 0.14^b^	10.00 ± 0.00	7.58 ± 0.14^abc^	10.00 ± 0.00	10.00 ± 0.00	7.67 ± 0.14^bc^	83.33 ± 0.58^bc^
Natural 2	7.83 ± 0.14^b^	7.58 ± 0.14^a^	7.50 ± 0.00^ab^	7.67 ± 0.14^b^	7.75 ± 0.25^b^	10.00 ± 0.00	7.75 ± 0.00^c^	10.00 ± 0.00	10.00 ± 0.00	7.75 ± 0.00^c^	83.83 ± 0.14^c^
Natural 3	7.50 ± 0.00^a^	7.42 ± 0.14^a^	7.50 ± 0.00^ab^	7.50 ± 0.00^ab^	7.50 ± 0.00^ab^	10.00 ± 0.00	7.42 ± 0.14^a^	10.00 ± 0.00	10.00 ± 0.00	7.50 ± 0.00^ab^	82.33 ± 0.14^a^
Wine	7.58 ± 0.14^a^	7.42 ± 0.14^a^	7.42 ± 0.14^a^	7.50 ± 0.00^ab^	7.58 ± 0.14^ab^	10.00 ± 0.00	7.42 ± 0.14^a^	10.00 ± 0.00	10.00 ± 0.00	7.42 ± 0.14^a^	82.33 ± 0.52^a^
Honey	7.58 ± 0.14^a^	7.67 ± 0.14^a^	7.50 ± 0.00^ab^	7.50 ± 0.00^ab^	7.58 ± 0.14^ab^	10.00 ± 0.00	7.67 ± 0.14^bc^	10.00 ± 0.00	10.00 ± 0.00	7.58 ± 0.14^abc^	83.08 ± 0.52^ab^
Semiwashed	7.50 ± 0.00^a^	7.58 ± 0.14^a^	7.50 ± 0.00^ab^	7.42 ± 0.14^a^	7.33 ± 0.14^a^	10.00 ± 0.00	7.50 ± 0.00^ab^	10.00 ± 0.00	10.00 ± 0.00	7.50 ± 0.00^ab^	82.33 ± 0.14^a^

Values followed by different superscripts in the same column show significantly different (*P* < 0.05). The values displayed in the table are the average and standard deviation of three replications. Natural 1, 2, and 3 samples were from different farmers. Natural 1, wine, honey, and semiwashed samples were from same farmer.

## Data Availability

All data obtained or analyzed from this study are included in this published article.

## Supplementary Materials

Supplementary Materials Table 1S of supplementary file reveals all individual volatile compounds and their classes identified in green and roasted Excelsa coffee powder prepared from diverse postharvest processes. GC-MS analysis detected 59 volatile compounds from 12 different classes in green beans and 85 volatile compounds from 18 classes in roasted beans.
