# Supplementary material for: Profile of Bioactive Compounds, Aromas, and Cup Quality of Excelsa Coffee (Coffea liberica var. dewevrei) Prepared from Diverse Postharvest Processes
Source: Int J Food Sci. 2022 Aug 18;2022:2365603. doi: 10.1155/2022/2365603 (PMC9410941; doi:10.1155/2022/2365603)
Supplement: Supplementary Materials — Table 1S of supplementary file reveals all individual volatile compounds and their classes identified in green and roasted Excelsa coffee powder prepared from diverse postharvest processes. GC-MS analysis detected 59 volatile compounds from 12 different classes in green beans and 85 volatile compounds from 18 classes in roasted beans. [file 2365603.f1.docx]

Table 1S Individual volatile compounds and their classes identified in green and roasted Excelsa coffee powder prepared from diverse post-harvest processes.

| LLRI | Compounds name | *Concentration in green beans (µg/kg beans) | | | | | | *Concentration in roasted beans (µg/kg beans) | | | | | |  |
| --- | --- | --- | --- | --- | --- | --- | --- | --- | --- | --- | --- | --- | --- | --- |
|  |  | Natural 1 | Natural 2 | Natural 3 | Wine | Honey | Semi-Washed | Natural 1 | Natural 2 | Natural 3 | Wine | Honey | Semi-Washed |  |
|  | **Aldehyde** |  |  |  |  |  |  |  |  |  |  |  |  |  |
| - | Acetaldehyde | 42.7 | 7.3 | 38.0 | 50.2 | 55.1 | 45.2 | nd | nd | nd | nd | nd | nd |  |
| - | Glycoaldehyde dimer (Hydroxy-acetaldehyde) | 70.5 | 94.0 | 64.4 | 96.2 | 96.3 | 69.9 | 1431.7 | 1553.8 | 1304.3 | 803.6 | 1782.2 | 1808.7 |  |
|  | **Total Aldehyde** | **113.2** | **101.2** | **102.4** | **146.4** | **151.4** | **115.1** | **1431.7** | **1553.9** | **1304.3** | **803.7** | **1782.2** | **1808.7** |  |
|  | **Alcohol** |  |  |  |  |  |  |  |  |  |  |  |  |  |
| - | Cyclobutanol | 25.0 | 43.4 | 34.0 | 35.2 | 49.4 | 25.1 | 905.6 | 874.9 | 922.9 | 507.0 | 1268.8 | 1079.3 |  |
| 1115 | 2-Butyloctanol | nd | nd | nd | nd | nd | nd | 45.4 | 36.7 | 43.7 | 24.7 | 20.6 | 37.2 |  |
| 1144 | 2-Ethyl-1-decanol | nd | nd | nd | nd | nd | nd | 68.4 | 39.6 | 28.2 | 46.2 | 13.9 | 21.2 |  |
| 1317 | 5-Methyl-2-hexanol | 7.8 | 22.3 | 19.1 | 24.3 | 11.0 | 8.3 | 159.0 | 167.0 | 144.8 | 97.5 | 211.2 | 214.8 |  |
| 1362 | 1-Hexanol | 3.56 | ND | 4.07 | 6.78 | 4.83 | 7.04 | nd | nd | nd | nd | nd | nd |  |
| 1456 | 1-Octen-3-ol | 2.7 | 1.5 | 2.8 | 2.8 | 3.6 | 2.6 | nd | nd | nd | nd | nd | nd |  |
| 1495 | 2-Butyl-1-Octanol | nd | nd | 0.7 | 1.4 | nd | nd | nd | nd | nd | nd | nd | nd |  |
| 1552 | Linalool | 1.8 | 0.8 | 0.9 | 0.9 | 0.8 | 0.9 | nd | nd | nd | nd | nd | nd |  |
| 1627 | 3-Methyl-2-hexanol | 5.0 | 2.0 | 2.0 | 2.5 | 1.8 | 0.5 | 771.0 | 467.2 | 597.6 | 69.4 | 445.1 | 738.6 |  |
| 1640 | Triethylene gylcol | 1.2 | 1.5 | 2.1 | 1.5 | 1.6 | 0.3 | nd | nd | nd | 27.1 | 46.9 | nd |  |
| 1650 | 3-Methyl-2-butanol | 0.94 | nd | nd | nd | nd | 0.4 | nd | nd | nd | nd | nd | nd |  |
| 1763 | 1-(2-Hydroxyethoxy)-2- (vinylthio)ethane | nd | nd | nd | nd | nd | nd | 16.11 | 47.5 | 12.2 | 18.4 | 28.7 | 25.5 |  |
| 1789 | 4-Methoxythio | nd | nd | nd | nd | nd | nd | 109.0 | 110.8 | 89.3 | 71.7 | 143.5 | 158.6 |  |
| 1899 | 2-Methoxyphenol | nd | nd | nd | nd | nd | nd | 49.0 | 48.7 | 45.3 | 28.8 | 60.1 | 59.1 |  |
|  | **Total Alcohol** | **47.6** | **71.5** | **65.7** | **75.5** | **70.5** | **45.0** | **2123.4** | **1792.6** | **1883.9** | **890.7** | **2215.4** | **2334.3** |  |
|  | **Carboxylic acid** |  |  |  |  |  |  |  |  |  |  |  |  |  |
| - | Glycoic acid (Hydroxy- Acetic acid) | 167.6 | 155.8 | 106.1 | 188.7 | 153.7 | 108.2 | 2977.5 | 3442.3 | 2758.2 | 1742.2 | 4105.5 | 3277.8 |  |
| 1461 | Acetic acid | 1.6 | 3.3 | 7.2 | 16.2 | 27.1 | 5.1 | 1203.4 | 1105.6 | 1090.6 | 783.0 | 2639.2.64 | 1729.5 |  |
| 1680 | Isovaleric acid | 15.0 | 34.9 | 37.8 | 67.9 | 90.3 | 62.2 | 2406.8 | 2423.0 | 3128.3 | 1426.3 | 3496.5 | 2905.3 |  |
|  | **Total Carboxylic acid** | **184.2** | **194.2** | **151.1** | **272.8** | **271.1** | **175.4** | **6587.7** | **6971.1** | **6978.2** | **3951.4** | **10241.1** | **7912.7** |  |
|  | **Ester** |  |  |  |  |  |  |  |  |  |  |  |  |  |
| - | Methyl isovalerate | 26.4 | 41.9 | 34.6 | 34.5 | 35.1 | 30.4 | 32.0 | 44.4 | 21.9 | 22.4 | 22.1 | 24.6 |  |
| - | Ethyl isovalerate | 102.4 | 128.4 | 103.8 | 226.5 | 130.2 | 109.6 | 217.8 | 211.8 | 228.9 | 148.8 | 296.0 | 266.9 |  |
|  | **Total Ester** | **128.8** | **170.3** | **138.4** | **262.0** | **165.3** | **140.1** | **249.8** | **256.3** | **250.8** | **171.3** | **318.2** | **291.5** |  |
|  | **Keton** |  |  |  |  |  |  |  |  |  |  |  |  |  |
| - | 3-Hexanone | 14.1 | 18.1 | 19.4 | 19.5 | 15.1 | 13.4 | 208.5 | 193.3 | 221.3 | 101.0 | 233.0 | 246.6 |  |
| - | 2,3-Pentanedione | nd | nd | nd | nd | nd | nd | 111.1 | 113.4 | 80.1 | 90.2 | 144.6 | 135.6 |  |
| 1216 | 2,2-Dimethylcyclobutanone | 27.3 | 26.3 | 26.6 | 38.6 | 33.3 | 17.3 | nd | nd | nd | nd | nd | nd |  |
| 1332 | Acetylcarbinol | nd | nd | nd | nd | nd | nd | 409.9 | 345.1 | 324.3 | 209.6 | 554.6 | 658.3 |  |
| 1476 | 1-Acetoxyacetone | nd | nd | nd | nd | nd | nd | 332.9 | 361.7 | 225.9 | 395.6 | 387.2 | 573.5 |  |
| 1541 | 2-Methyl-3-pentanone | nd | nd | nd | nd | nd | nd | 168.1 | 334.5 | 167.3 | 54.4 | 272.1 | 183.5 |  |
| 1542 | 3,3-Dimethylbutanone | nd | nd | nd | nd | nd | nd | 117.4 | 107.9 | 72.6 | 36.9 | 129.6 | 113.6 |  |
| 1543 | 1-Acetoxy-2-butanone | nd | nd | nd | nd | nd | nd | 95.6 | 113.9 | 88.1 | 45.9 | 104.3 | 96.9 |  |
| 1879 | 6-Methyl-5-Octen-2-one | nd | nd | nd | nd | nd | nd | 52.2 | 55.5 | 56.7 | 37.6 | 59.5 | 59.0 |  |
|  | **Total Keton** | **41.43** | **44.37** | **45.95** | **58.11** | **48.40** | **30.71** | **1495.6** | **1625.4** | **1236.3** | **999.3** | **1967.5** | **2187.6** |  |
|  | **Alkana** |  |  |  |  |  |  |  |  |  |  |  |  |  |
| 1134 | 2,6,10-Trimethyl-Tetradecane, | nd | nd | nd | nd | nd | 15.11 | nd | nd | nd | nd | nd | nd |  |
| 1135 | 3,6-Dimethyl-decane | 18.1 | 26.2 | 23.4 | 30.8 | 27.7 | ND | nd | nd | nd | nd | nd | nd |  |
| 1136 | Hexadecane | nd | nd | nd | nd | nd | 29.5 | nd | nd | nd | nd | nd | nd |  |
| 1146 | 2,4-Dimethyl-heptane | 10.7 | 13.5 | 12.7 | 14.0 | 10.7 | 11.5 | nd | nd | nd | nd | nd | nd |  |
| 1159 | Dodecane | nd | nd | nd | nd | nd | nd | 29.2 | 35.5 | 34.8 | ND | 16.4 | 19.9 |  |
| 1203 | 2,4-Dimethyl-undecane | 4.6 | 6.9 | 7.2 | 8.6 | 5.2 | 4.8 | nd | nd | nd | nd | nd | nd |  |
| 1248 | 3,5-Dimethyl-heptane | 17.3 | 23.0 | 27.4 | 29.5 | 21.4 | 15.9 | nd | nd | nd | nd | nd | nd |  |
| 1308 | 3-Methyl-Tridecane | 5.06 | 7.66 | nd | nd | 7.88 | 3.79 | nd | nd | nd | nd | nd | nd |  |
| 1336 | Nonane | 5.28 | nd | 9.39 | 11.60 | 8.31 | 3.52 | nd | nd | nd | nd | nd | nd |  |
| 1355 | 3,5-Dimethyl-octane | nd | nd | 6.15 | 6.01 | nd | nd | nd | nd | nd | nd | nd | nd |  |
| 1366 | 2,4,6-Trimethyl-octane | 3.2 | 5.6 | 5.0 | 3.4 | 7.0 | 3.0 | nd | nd | nd | nd | nd | nd |  |
| 1370 | 2,9-Dimethyl-undecane | 3.3 | 2.2 | 3.2 | 5.1 | 2.7 | 1.7 | nd | nd | nd | nd | nd | nd |  |
| 1386 | 3-Methyl-Dodecane | 2.4 | 4.5 | 7.1 | 7.9 | 4.9 | 4.1 | nd | nd | nd | nd | nd | nd |  |
| 1401 | 2,3,5-trimethyl-decane | 2.2 | 3.1 | 5.1 | 5.2 | 4.0 | 1.7 | nd | nd | nd | nd | nd | nd |  |
| 1514 | 5,7-Dimethyl-undecane | nd | 0.6 | 0.9 | 1.5 | nd | nd | nd | nd | nd | nd | nd | nd |  |
| 1530 | α-Copaene | nd | nd | 0.4 | 1.0 | nd | nd | nd | nd | nd | nd | nd | nd |  |
| 1660 | 1-Ethoxy-, (Z)-2-heptene, | nd | 1.3 | 0.7 | 4.0 | 1.2 | nd | nd | nd | nd | nd | nd | nd |  |
| 2026 | Eicosane | 1.5 | 0.8 | 0.5 | 0.9 | 0.4 | nd | nd | nd | nd | nd | nd | nd |  |
| 2105 | 1-Ethylthio-1,3-Butadiene | nd | nd | nd | nd | nd | nd | 89.9 | 99.3 | 104.1 | 52.3 | 118.1 | 113.5 |  |
|  | **Total Alkana** | **73.6** | **89.7** | **109.1** | **127.4** | **95.6** | **72.2** | **119.2** | **134.8** | **138.9** | **52.3** | **134.5** | **133.4** |  |
|  | **Pyridine** |  |  |  |  |  |  |  |  |  |  |  |  |  |
| 1217 | Pyridine | nd | nd | nd | nd | nd | nd | 1212.8 | 1454.6 | 1262.7 | 767.7 | 1343.7 | 1334.2 |  |
| 1335 | 3-Methyl-pyridine | nd | nd | nd | nd | nd | nd | 212.9 | 300.8 | 204.6 | 173.6 | 400.1 | 261.0 |  |
| 1425 | 4-Ethyl-pyridine | 5.5 | 5.4 | 5.1 | 2.8 | 6.3 | 4.7 | 475.5 | 539.8 | 509.2 | 291.4 | 504.2 | 517.2 |  |
| 1437 | 2,6-Dimethyl-4-pyridinamine | 2.4 | 1.7 | 5.6 | 5.9 | 3.7 | 2.5 | 154.3 | 110.6 | 196.4 | 173.2 | 195.2 | 189.4 |  |
| 1515 | 4-Ethenyl-pyridine, | nd | nd | nd | nd | nd | nd | 98.5 | 86.6 | 71.6 | 61.4 | 112.9 | 123.6 |  |
| 1593 | 5-Ethenyl-2-methyl-pyridine | nd | nd | nd | nd | nd | nd | 29.7 | 32.8 | 29.9 | 24.6 | 32.5 | 31.0 |  |
| 1650 | 2-Acetylpyridine | nd | nd | nd | nd | nd | nd | 30.5 | 28.6 | 39.1 | 30.8 | 55.1 | 47.2 |  |
| 1655 | 2,6-Dimethyl-3-pyridinol | nd | nd | nd | nd | nd | nd | 35.1 | 44.4 | 42.4 | 31.2 | 78.4 | 39.0 |  |
| 1811 | 3-Acetoxypyridine | nd | nd | nd | nd | nd | nd | 186.6 | 188.4 | 173.4 | 118.7 | 246.2 | 270.5 |  |
| 2091 | 3-Isobutylpyridine | nd | nd | nd | nd | nd | nd | 65.3 | 85.7 | 68.8 | 40.5 | 56.9 | 89.6 |  |
|  | **Total Pyridine** | **7.85** | **10.77** | **7.15** | **8.67** | **9.99** | **7.24** | **2501.2** | **2872.5** | **2595.9** | **1713.0** | **3025.1** | **2902.7** |  |
|  | **Pyrimidine derivative** |  |  |  |  |  |  |  |  |  |  |  |  |  |
| 1241 | 1,3-Diazine | nd | nd | nd | nd | nd | nd | 56.5 | 62.3 | 37.7 | nd | 56.8 | 78.3 |  |
| 1365 | 4,6-Dimethyl-pyrimidine | nd | nd | nd | nd | nd | nd | 329.8 | 274.4 | 280.4 | 146.6 | 426 | 485.1 |  |
|  | **Total Pyrimidine derivative** | **0.00** | **0.00** | **0.00** | **0.00** | **0.00** | **0.00** | **386.3** | **336.6** | **318.1** | **146.6** | **454.3** | **563.4** |  |
|  | **Furan** |  |  |  |  |  |  |  |  |  |  |  |  |  |
| 1251 | 2-(Methoxymethyl)-furan | nd | nd | nd | nd | nd | nd | 81.4 | 85.9 | 87.6 | 87.8 | 152.3 | 90.2 |  |
| 1290 | Dihydro-2-methyl-3-furanone | 4.8 | 9.0 | 10.3 | 8.6 | 7.6 | 4.7 | 157.8 | 72.4 | 216.0 | 109.2 | 214.2 | 221.1 |  |
| 1404 | Tetrahydro-3-Furanol | nd | nd | nd | nd | nd | nd | 58.9 | 63.8 | 53.8 | 40.4 | 77.4 | 80.5 |  |
| 1490 | Furfural | 1.4 | 1.8 | 2.3 | 2.6 | 2.9 | 1.8 | 1092.8 | 1000.4 | 972.7 | 649.0 | 1566.7 | 1604.7 |  |
| 1513 | 2-Furylmethyl formate | nd | nd | nd | nd | nd | nd | 40.6 | 73.4 | 49.3 | 33.1 | 63.6 | 67.2 |  |
| 1539 | 2-Acetylfuran | nd | nd | nd | nd | nd | nd | 65.1 | 98.2 | 50.5 | 96.2 | 60.8 | 156.7 |  |
| 1552 | 2-Acetoxymethylfuran | 1.1 | 2.2 | 0.6 | 0.6 | 1.9 | 1.0 | 438.5 | 397.7 | 391.6 | 291.7 | 583.2 | 538.7 |  |
| 1606 | 5-Methylfurfural | 0.8 | 0.9 | 1.4 | 2.1 | 1.5 | 0.6 | 154.7 | 377.3 | 167.7 | 543.4 | 553.5 | 439.8 |  |
| 1652 | Tetramethylfuran | nd | nd | nd | nd | nd | nd | 63.6 | 61.0 | 52.6 | 23.0 | 58.6 | 54.3 |  |
| 1662 | Tetrahydro-2,5-dimethyl-cis-furan, | nd | nd | nd | nd | nd | 0.6 | nd | nd | nd | nd | nd | nd |  |
| 1678 | 2-Furanmethanol | nd | nd | nd | nd | nd | nd | 900.6 | 1003.0 | 158.5 | 652.1 | 1784.3 | 1793.6 |  |
| 1712 | Furfuryl pentanoate | nd | nd | nd | nd | nd | nd | 34.0 | 38.3 | 39.2 | 23.6 | 39.5 | 46.2 |  |
| 1775 | 3,4-Dimethyl-2,5-Furandione | nd | nd | nd | nd | nd | nd | 93.9 | 95.2 | 99.0 | 29.8 | 75.4 | 116.7 |  |
| 2058 | Furaneol | nd | nd | nd | nd | nd | nd | 102.7 | 119.7 | 107.9 | 56.0 | 125.8 | 140.7 |  |
|  | **Total Furan** | **3.3** | **4.3** | **4.8** | **5.3** | **6.3** | **3.7** | **3284.7** | **3586.3** | **2446.5** | **2635.3** | **5335.2** | **536.0** |  |
|  | **Pyrazine** |  |  |  |  |  |  |  |  |  |  |  |  |  |
| 1297 | Methyl-pyrazine | 5.6 | 6.0 | 19.5 | 13.8 | 10.5 | 7.0 | 1088.7 | 1054.4 | 934.6 | 561.7 | 1417.8 | 1264.8 |  |
| 1359 | 2,5-Dimethyl-pyrazine | 4.6 | 8.0 | 6.8 | 6.4 | 7.2 | 4.3 | 314.2 | 198.7 | 342.5 | 172.1 | 469.9 | 386.2 |  |
| 1361 | 2,6-Dimethyl-pyrazine | 4.4 | 7.6 | 6.3 | 6.4 | 9.1 | 3.0 | 829.1 | 892.7 | 684.0 | 377.4 | 915.2 | 781.4 |  |
| 1369 | Ethyl-pyrazine | nd | nd | nd | nd | nd | nd | 139.9 | 132.9 | 217.0 | 113.2 | 283.2 | 202.7 |  |
| 1381 | 2,3-Dimethyl-pyrazine | nd | nd | nd | nd | nd | nd | 269.3 | 274.3 | 256.1 | 156.7 | 363.9 | 306.5 |  |
| 1419 | 2-Ethyl-5-methyl-pyrazine | 2.2 | 2.3 | 4.2 | 4.7 | 3.9 | 3.2 | 443.8 | 347.0 | 423.45 | 189.5 | 506.9 | 415.2 |  |
| 1426 | 2-Ethyl-6-methyl-pyrazine | nd | nd | nd | nd | nd | nd | 181.9 | 162.2 | 182.0 | 98.9 | 242.0 | 269.0 |  |
| 1440 | Trimethyl-pyrazine, | nd | nd | nd | nd | nd | nd | 377.7 | 395.9 | 336.0 | 110.1 | 454.7 | 387.1 |  |
| 1476 | 3-Ethyl-2,5-dimethyl-Pyrazine | 1.2 | 2.7 | 2.6 | 3.6 | 2.6 | 1.3 | 617.0 | 577.4 | 610.5 | 162.5 | 668.0 | 520.9 |  |
| 1546 | 3,5-Diethyl-2- methyl-pyrazine | nd | nd | 0.2 | 0.4 | nd | nd | 136.3 | 69.8 | 87.7 | 50.6 | 88.2 | 57.1 |  |
| 1573 | 2-Isopropenyl-3-methylpyrazine | nd | nd | nd | nd | nd | nd | 53.4 | 53.2 | 50.1 | 45.0 | 51.1 | 57.8 |  |
| 1705 | 1-Methylethenyl-pyrazine, | nd | nd | nd | nd | nd | nd | 87.8 | 80.2 | 85.2 | 23.6 | 85.1 | 82.8 |  |
| 1726 | 2-Ethyl-3-methoxy-pyrazine | nd | nd | nd | nd | nd | nd | 31.78 | 49.6 | 34.6 | 30.8 | 66.2 | 53.7 |  |
| 1730 | Pyrazine, 3-acetyl-2-methyl | nd | nd | nd | nd | nd | nd | 130.7 | 107.8 | 117.6 | 60.5 | 128.3 | 136.6 |  |
| 1741 | 2-Acetyl-6-methyl pyrazine | nd | nd | nd | nd | nd | nd | 197.5 | 192.5 | 193.2 | 97.1 | 241.2 | 203.8 |  |
| 1747 | Pyrazinamide | nd | nd | nd | nd | nd | nd | 112.0 | 70.9 | 100.0 | 73.7 | 168.2 | 158.5 |  |
| 1775 | 2-Methyl-5-propenyl-, (E)-pyrazine | nd | nd | nd | nd | nd | nd | 23.1 | 25.3 | 27.7 | 29.1 | 42.43 | 31.3 |  |
|  | **Total Pyrazine** | **18.0** | **39.6** | **26.5** | **33.2** | **33.2** | **18.7** | **5033.9** | **4684.9** | **4682.2** | **2352.4** | **6192.4** | **5315.1** |  |
|  | **Pyrazole** |  |  |  |  |  |  |  |  |  |  |  |  |  |
| 1493 | 3,5-Dimethyl-1-allyl-pyrazole | 1.0 | 1.2 | 1.2 | 1.0 | 1.9 | 1.1 | nd | nd | nd | nd | nd | nd |  |
| 1540 | 4-Acetylpyrazole | nd | nd | nd | nd | nd | nd | 167.8 | 220.4 | 116.9 | 131.1 | 164.1 | 278.4 |  |
|  | **Total Pyrazole** | **1.0** | **1.2** | **1.2** | **1.0** | **1.9** | **1.1** | **167.8** | **220.4** | **116.9** | **131.1** | **164.1** | **278.4** |  |
|  | **Benzoxazine** |  |  |  |  |  |  |  |  |  |  |  |  |  |
| 1524 | 2H-1,4-Benzoxazin-3(4H)-one | nd | nd | nd | nd | nd | nd | 54.5 | 67.3 | 81.6 | 32.0 | 120.8 | 109.9 |  |
|  | **Total Benzoxazine** | **0.0** | **0.0** | **0.0** | **0.0** | **0.0** | **0.0** | **54.5** | **67.3** | **81.6** | **32.0** | **120.8** | **109.9** |  |
|  | **Hydrazine** |  |  |  |  |  |  |  |  |  |  |  |  |  |
| 1578 | 1,1-Diethylhydrazine | nd | nd | 0.8 | nd | nd | nd | 20.4 | 21.6 | 23.2 | ND | 112.2 | 21.2 |  |
|  | **Total Hydrazine** | **0.0** | **0.0** | **0.8** | **0.0** | **0.0** | **0.0** | **20.4** | **21.6** | **23.2** | **0,00** | **112.2** | **21.2** |  |
|  | **Pyrrole** |  |  |  |  |  |  |  |  |  |  |  |  |  |
| 1669 | 2-Formyl-5-methylpyrrole | nd | nd | nd | nd | nd | nd | 186.1 | 121.9 | 57.8 | 88.7 | 250.6 | 27 |  |
| 1697 | 2-Acetyl-1-methylpyrrole | nd | nd | nd | nd | nd | nd | 81.3 | 99.2 | 82.8 | 55.4 | 103.7 | 115.3 |  |
| 2008 | 2-Acetylpyrrole | nd | nd | nd | nd | nd | nd | 88.8 | 95.9 | 100.3 | 57.7 | 121.0 | 128.4 |  |
| 2065 | 2-Formylpyrrole | nd | nd | nd | nd | nd | nd | 97.7 | 102.0 | 98.3 | 55.9 | 113.0 | 127.5 |  |
|  | **Total Pyrrole** | **0.0** | **0.0** | **0.0** | **0.0** | **0.0** | **0.0** | **454.0** | **419.0** | **339.1** | **257.6** | **588.2** | **640.9** |  |
|  | **Thiophene** |  |  |  |  |  |  |  |  |  |  |  |  |  |
| 1978 | 2-Thiophenemethanol | nd | nd | nd | nd | nd | nd | 37.6 | 51.5 | 49.0 | 25.7 | 50.6 | 68.7 |  |
|  | **Total Thiophene** | **0.0** | **0.0** | **0.0** | **0.0** | **0.0** | **0.0** | **37.6** | **51.5** | **49.0** | **25.7** | **50.7** | **68.7** |  |
|  | **Pyrone** |  |  |  |  |  |  |  |  |  |  |  |  |  |
| 2017 | Maltol | nd | nd | nd | nd | nd | nd | 168.6 | 182.7 | 180.6 | 109.6 | 185.8 | 206.7 |  |
| 2294 | Pyranone | nd | nd | nd | nd | nd | nd | 34.7 | 59.7 | 65.5 | 28.6 | 82.7 | 120.6 |  |
|  | **Total Pyrone** | **0.0** | **0.0** | **0.0** | **0.0** | **0.0** | **0.0** | **203.3** | **242.4** | **246.1** | **138.2** | **268.4** | **327.3** |  |
|  |  |  |  |  |  |  |  |  |  |  |  |  |  |  |
|  | **Quinolone** |  |  |  |  |  |  |  |  |  |  |  |  |  |
| 2129 | 4-Methyl-2-quinolone | nd | nd | nd | nd | nd | nd | 64.8 | 86.5 | 85.3 | 42.5 | 73.6 | 108.0 |  |
|  | **Total Quinolone** | **0.0** | **0.0** | **0.0** | **0.0** | **0.0** | **0.0** | **64.8** | **86.5** | **85.2** | **42.5** | **73.6** | **108.0** |  |
|  | **Benzene and derivatives** |  |  |  |  |  |  |  |  |  |  |  |  |  |
| 1422 | Methoxymethyl-benzene | nd | 1.5 | 3.7 | 2.7 | nd | nd | nd | nd | nd | nd | nd | nd |  |
| 1522 | 2-Methoxyethyl-benzene | 0.8 | 0.5 | 1.4 | 2.3 | 1.2 | 0.8 | nd | nd | nd | nd | nd | nd |  |
| 1550 | 2-methyl-1,4-benzenediol | nd | 0.3 | 0.30 | 0.6 | 0.4 | nd | nd | nd | nd | nd | nd | nd |  |
| 1566 | Benzaldehyde | 0.6 | 0.8 | 0.8 | 2.3 | 7.6 | 0.6 | 34.4 | 26.2 | 37.7 | 19.9 | 48.2 | 47.8 |  |
| 1717 | 4-Propoxyaniline | 0.4 | 0.7 | 0.5 | 1.2 | 0.6 | 0.4 | 29.6 | 32.0 | 27.4 | 18.7 | 31.8 | 35.6 |  |
| 1829 | Methyl salicylate | 4.4 | 5.3 | 3.3 | 9.5 | 6.4 | 2.0 | 226.4 | 264.9 | 257.8 | 144.1 | 304.9 | 248.8 |  |
| 1861 | Ethyl salicylate | 18.3 | 26.3 | 11.9 | 29.1 | 17.1 | 4.3 | 407.8 | 480.8 | 371.3 | 247.0 | 462.9 | 317.6 |  |
| 1908 | Benzyl Alcohol | 2.6 | 1.8 | 1.9 | 4.2 | 1.8 | 1.1 | 159.1 | 162.9 | 147.1 | 99.7 | 184.3 | 206.0 |  |
| 1949 | Phenylethyl Alcohol | 2.1 | 1.4 | 2.0 | 3.9 | 2.8 | 1.6 | 71.5 | 74.3 | 77.0 | 46.6 | 66.7 | 67.7 |  |
| 2216 | 4-Vinylguaiacol | nd | nd | nd | nd | nd | nd | 148.0 | 158.9 | 142.4 | 76.3 | 213.0 | 225.8 |  |
|  | **Total Benzena** | **29.1** | **37.8** | **25.9** | **55.2** | **37.7** | **10.9** | **1076.9** | **1199.9** | **1060.7** | **652.3** | **1311.7** | **1149.3** |  |

LRI = Linear Retention Index. nd = not detected. Natural 1, 2, and 3 samples were from different farmers. Natural 1, wine, honey, and semi-washed samples were from same farmer. *The relative concentration of volatile compounds was calculated by comparing the peak area of ​​the specific compound in the sample with the peak area of ​​ internal standard (3-heptanone). The values displayed in the table are average of two replications.
